# Patterns of Herbivory in Neotropical Forest Katydids as Revealed by DNA Barcoding of Digestive Tract Contents

**DOI:** 10.3390/d14020152

**Published:** 2022-02-21

**Authors:** Christine M. Palmer, Nicole L. Wershoven, Sharon J. Martinson, Hannah M. ter Hofstede, W. John Kress, Laurel B. Symes

**Affiliations:** 1 Natural Sciences Department, Castleton University, 233 South Street, Castleton, VT 05735, USA; 2 Department of Biological Sciences, Dartmouth College, 78 College Street, Hanover, NH 03755, USA; 3 Smithsonian Tropical Research Institute, Balboa, Ancón, Apartado 0843-03092, Panama; 4 K. Lisa Yang Center for Conservation Bioacoustics, Cornell Lab of Ornithology, Cornell University, 159 Sapsucker Woods Road, Ithaca, NY 14850, USA; 5 Graduate Program in Ecology, Evolution, Environment and Society, Dartmouth College, 64 College Street, Suite 102, Hanover, NH 03755, USA; 6 Department of Botany, National Museum of Natural History, Smithsonian Institution, P.O. Box 37012, Washington, DC 20013, USA

**Keywords:** trophic interactions, diet specialization, DNA barcoding, bush cricket, Barro Colorado Island, Panama, katydid, tropical trees

## Abstract

Many well-studied animal species use conspicuous, repetitive signals that attract both mates and predators. Orthopterans (crickets, katydids, and grasshoppers) are renowned for their acoustic signals. In Neotropical forests, however, many katydid species produce extremely short signals, totaling only a few seconds of sound per night, likely in response to predation by acoustically orienting predators. The rare signals of these katydid species raises the question of how they find conspecific mates in a structurally complex rainforest. While acoustic mechanisms, such as duetting, likely facilitate mate finding, we test the hypothesis that mate finding is further facilitated by colocalization on particular host plant species. DNA barcoding allows us to identify recently consumed plants from katydid stomach contents. We use DNA barcoding to test the prediction that katydids of the same species will have closely related plant species in their stomach. We do not find evidence for dietary specialization. Instead, katydids consumed a wide mix of plants within and across the flowering plants (27 species in 22 genera, 16 families, and 12 orders) with particular representation in the orders Fabales and Laurales. Some evidence indicates that katydids may gather on plants during a narrow window of rapid leaf out, but additional investigations are required to determine whether katydid mate finding is facilitated by gathering at transient food resources.

## 1. Introduction

Animals experience intense pressure to find food and mates while avoiding predation [1-3]. For many species, mate finding relies on signals that allow one sex to locate the other, and can integrate a variety of sensory modalities including visual, acoustic, electrical, and olfactory channels [4]. The same signals that increase detectability by mates can increase detectability by predators as well, and predators can impose intense selection both on the individuals doing the signaling, as well as on the individuals who are searching for mates [5-8]. Consequently, there is often intense selection for strategies that facilitate mate finding while minimizing exposure to predation.

Many Orthopterans, including crickets, katydids, and grasshoppers, are known for their conspicuous signals [9,10]. These species often use energetically expensive acoustic signals to attract mates [11,12], with signals repeated again and again for a large portion of the day or night. Orthopteran signals often attract predators as well [13,14]. In the Neotropics, eavesdropping gleaning bats such as *Trachops cirrhosis* and *Lophostoma silvicolum* hunt katydids and other small animals by eavesdropping on the sounds that they produce [15-17]. Likely as a result of this acoustically targeted predation, many Neotropical forest katydids produce vanishingly little sound. In a survey of 16 phaneropterine katydid species (Tettigoniidae), none produced more than five seconds of sound per night, with most species emitting infrequent calls of 20–200 ms in duration [18]. Many of these katydid species have calls with carrier frequencies that are in the high audible or ultrasonic range [19], characteristics that would also cause a call to attenuate quickly [20], particularly in dense vegetation. Beyond Phaneropterinae, other katydid subfamilies also have instances of low calling rates in Neotropical forests, with some conocephaline and pseudophylline species producing less than 30 s of sound per night [21]. While many Neotropical forest katydids produce little sound, there are examples of species that call substantially more, such as *Ischnomela pulchripennis*, a species that is associated with spiny bromeliads that provide protection from predator attack [17]. However, with key exceptions [22], relatively little is known about possible associations between particular species of katydids and their host plants and how those associations may interact with signal structure or signaling strategy.

For species that produce so few acoustic signals, one of the inescapable questions is how they are able to encounter mates in a dense and structurally complex rainforest. There are multiple mechanisms that could facilitate mate finding in species that produce only seconds of long-distance advertisement signal each night. Phaneropterine katydids engage in mating duets, where the female replies to the male signal with a short tick, providing information about her presence and receptivity (reviewed in [23]). Duetting alone may be enough to allow katydids to find each other, if the female replies incentivize short-term risk taking and elevate male signaling rate. Males will also produce ticks that resemble the female reply, likely as a competitive mechanism that confounds interception by other males [24]. If males are producing sounds to jam other males, it suggests that it is common for multiple individuals to be present and interacting during mating, again reflecting effective strategies for co-localizing with conspecifics, rather than rare chance encounters between pairs in the forest.

An additional mechanism that could further facilitate mate encounter while minimizing conspicuousness to predators is host plant specificity. In some species, animals mate on or near their food resources, streamlining the encounter process [25,26]. Animals that find mates on a food resource can reduce travel time and associated predation risk, and species that gather at food resources may also be able to use less conspicuous signals that enable them to compete for nearby mates without attracting distant predators. If katydids gather on particular host plants and search for mates where they are gathered, this food-based aggregation strategy could dramatically lower the hurdle to mate finding, reducing female travel costs and predation risk and allowing effective pairing, even with rare, short duration, rapidly attenuating signals.

Host plant specialization provides opportunities and challenges. Mature tropical forests contain a diversity of vegetation, much of which is heavily protected by secondary compounds and chemically defended against most herbivores [27-29]. Herbivores respond to plant defenses with a diversity of strategies, including extreme host generalization, where they eat small quantities of many plants to minimize the impacts of each type of toxin [30,31], or host plant specialization, where they evolve to tolerate or even repurpose a particular type of chemical defense [32,33]. In most habitats, herbivorous Orthopterans consume a wide range of plants [34]. However, there are cases where Orthopterans specialize on a particular food source, with some displaying strong associations and genetic differentiation based on diet [35,36], while others demonstrate strong preferences for specific plants but accept other plant species when preferred options are not available [37].

We test the hypothesis that many Neotropical forest phaneropterines are host plant specialists, facilitating pairing and reducing the demands associated with eating a large diversity of highly defended rainforest plants. The diet specialization hypothesis predicts that all katydids of a particular species will consistently consume the same or closely related plant species across space and time. An alternative hypothesis is that katydids are more generalized in their diets, a hypothesis that would be supported by sampling multiple individuals of the same katydid species and finding that they had been eating taxonomically diverse plant species.

Katydids are renowned for their camouflage and finding them in the forest can be very challenging, particularly because many species occur in the forest canopy [38]. Because it is so difficult to observe these animals in the wild, it is also difficult to determine if they are dietary specialists. A detailed review of the literature on these katydid species [19] did not yield any published records of diet. Fortunately, DNA sequencing approaches make it possible to identify plants that are part of the diets by extracting plant material from the stomachs of captured katydids and amplifying plant DNA barcodes [39-42]. These plant DNA barcodes have been developed specifically for the research site on Barro Colorado Island in Panama and have been previously utilized to generate a community phylogeny of trees and assign plant species to fine roots collected in the soil [43,44]. Here we employ these barcodes to test diet specialization of katydids.

## 2. Materials and Methods

### 2.1. Katydid Capture and Sample Preparation

For gut content characterization, katydids (family Tettigoniidae, primarily Phaneropterinae) were captured at building lights on Barro Colorado Island, Panama (9.16491, −79.83734). Katydids were collected during nighttime hours between late December and early March of 2015–2018, with 82% of the samples collected between 29 December and 31 January. During this season, katydids are actively calling and mating. Lights were checked two times per night, at approximately 23:00 and 04:30. When analyses were reported by year, the data are grouped by collecting season (e.g., ‘2017’ represents data collected from late December of 2016 to early March of 2017). For each katydid, we recorded the location of the light and time of capture, then immediately froze the insect to interrupt digestion. Katydids were identified to species in accordance with available resources [19,45], with published morphospecies names used to identify three species. Light trapping captured many different katydid species, with relatively even representation across species, rather than a few dominant species. In the current study, we focused on the most commonly captured species in order to represent multiple individuals of the same species. Plant DNA was obtained by dissecting the katydid and isolating the digestive tract, which was placed in a microcentrifuge tube for DNA extraction [40].

### 2.2. DNA Extraction and Amplification

DNA was purified and amplified as described in Symes et al. [40]. Briefly, DNA was purified from dissected digestive tracts following the manufacturer’s instructions using the QIAGEN DNeasy Plant Mini Kit (Qiagen 69104; Qiagen, Hilden, Germany) with the following modification. After initial homogenization with micropestles, 50 μL of AP1 buffer was added and the sample was further crushed before adding the 350 μL of AP1 buffer. Primers were utilized to amplify three conserved regions of the plastid genome following the procedure described in [43] (Table S1). Of the three regions, the *rbcL* region has the highest sequence conservation across plant species and is easy to sequence, the *psbA/trnH* region is intermediate in variability, but sometimes difficult to interpret, and the *matK* region is highly variable, but often difficult to amplify. PCR reactions were prepared using 5 μL template, 20 μL GoTaq Green PCR mastermix (Promega M7122; Madison, WI, USA), 0.5 μM F and 0.5 μM R primer, and water to a final volume of 40 μL. Thermocycler (ABI Veriti 96-well, model 9902) conditions for *rbcLa* and *psbA-trnH* were: 95 °C for 3 min, 35 cycles (95 °C for 30 s, 55 °C for 30 s, 72 °C for 1 min), 72 °C for 10 min and for *matK* were: 95 °C for 3 min, 40 cycles (95 °C for 30 s, 50 °C for 30 s, 72 °C for 1 min), 72 °C for 10 min. Samples were analyzed with *rbcLa* and *psbA-trnH* primers first, and then PCR was conducted with *matK* primers if needed to achieve a minimum of two successful primer sets per sample.

For positive samples, the remaining 30 μL of PCR product was purified using a Wizard PCR Clean-Up System (Promega A9281; Madison, WI, USA) and resuspended in 50 μL nuclease-free water. 2 μL of purified PCR product was mixed with 25 ng of the appropriate forward primer for the amplicon (*rbc*, rbc_SI_For; *psb*, psbA3’f; *matK*, matKfor_KIM3F) in a 15 μL reaction volume and sequenced using Sanger sequencing through Genewiz (Genewiz, South Plainfield, NJ, USA). Sequences were trimmed using Geneious and a 0.1 error probability limit. Following editing, sequences were exported in FASTA format for analysis with BLAST. Sequence data is available in FASTA format with the Supplementary Materials.

### 2.3. Identification of Plant Species

Sequences were assigned to plant species using the BLAST tool in Geneious based on identity similarity and query coverage between sequences from the gut samples and a custom reference database containing BCI-specific plant barcodes (*rbcL, psbA/trnH, matK*) for trees ([43]), shrubs, and lianas (Kress, unpubl). (Table S2) For samples with successful amplification for two or three of the gene markers, plant species identification was assigned if there was a match for the highest sequence similarity across multiple gene markers. If there were multiple taxa with the same similarity, identification was made to genus or family level. If there was no overlap between two or three gene markers, the sample was labeled as “Conflict”, indicating that different primer sets match different plant taxa. For samples with successful amplifications for only one gene marker, plant species identification was made to the species with the highest percent identity and query coverage. If more than one plant had similar identity and coverage, sequences were assigned to the level of concordance in the cluster (genus or family). Data visualizations were created with Microsoft Excel and Powerpoint.

### 2.4. Growth Height Assignment

Plants with species- or genus-level identification were assigned a growth height category based on the “plant growth form” data in the relevant overview page for each plant species from the Encyclopedia of Life [46]. Plants were binned into the following categories with the maximum height listed: Shrub (6 m), Understory tree (Tree 15 m, Tree 20 m, Tree 25 m), and Canopy Tree (Tree 30 m, Tree 40 m, Tree 70 m). In one case (genus *Ocotea*), genus-level identification was included because all species in that genus fell in the same growth category.

## 3. Results

**Figure 1.**
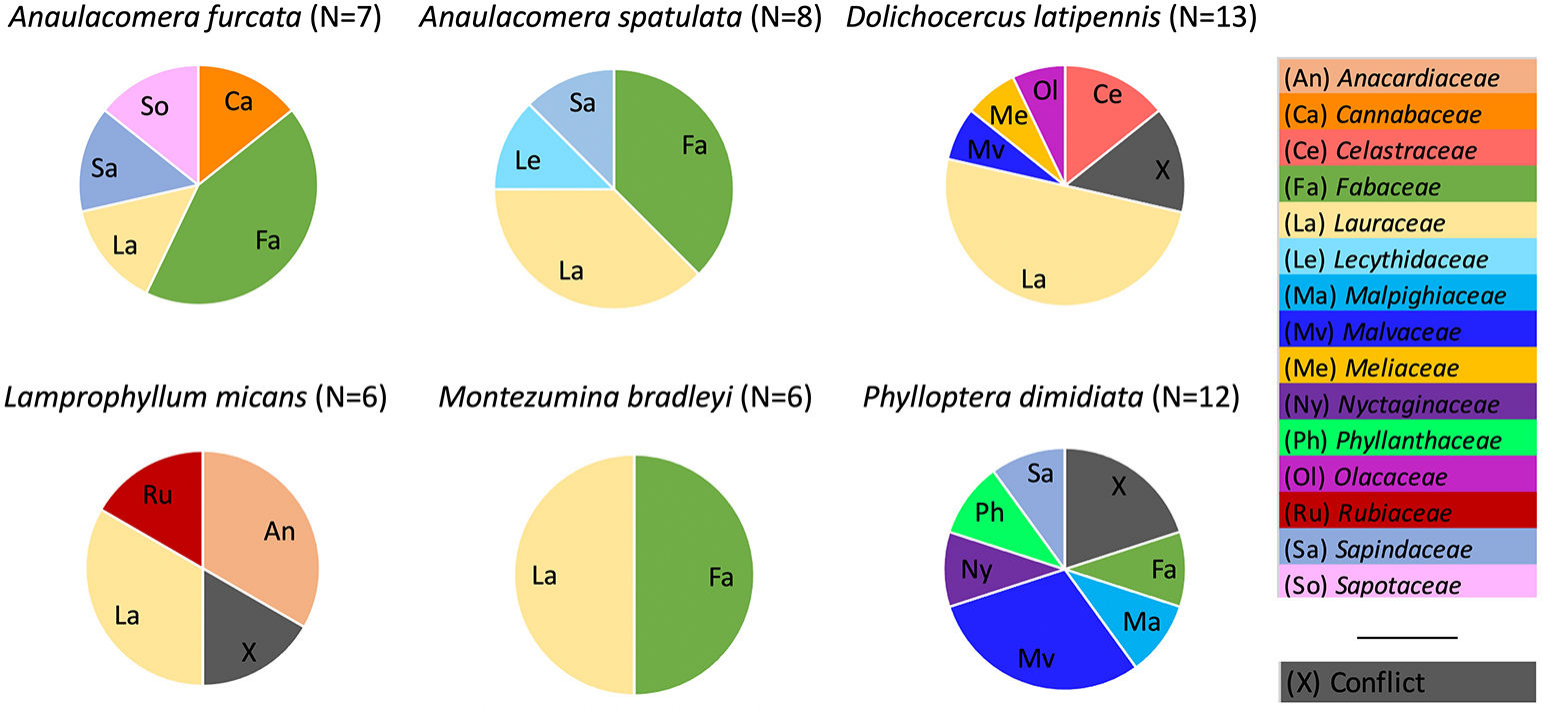
Plant families recovered from the stomach contents of six common katydid species on Barro Colorado Island, Panama. “Conflict” indicates that different primer sets show different plant species identifications for a given individual katydid.

**Figure 2.**
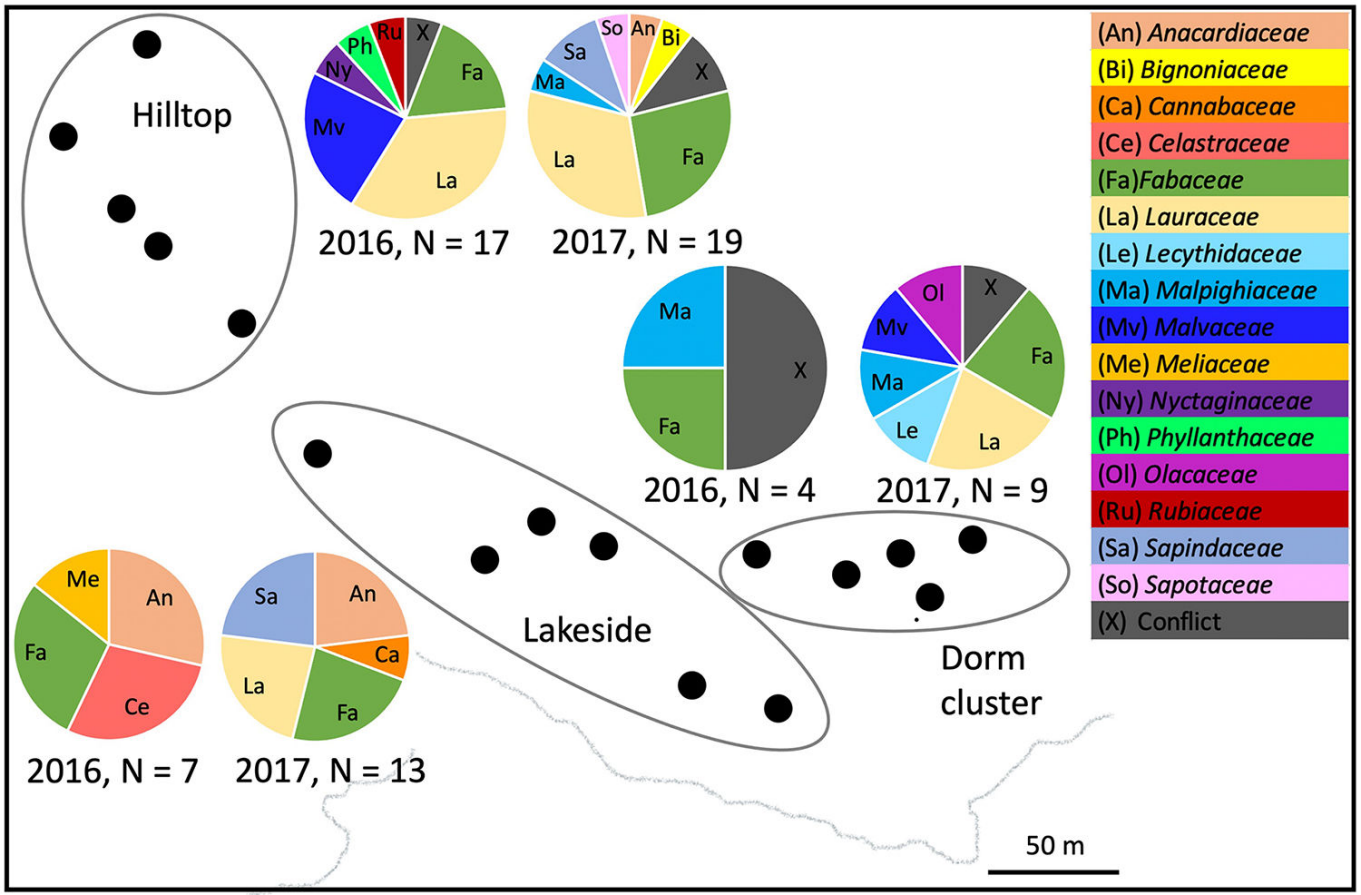
Map of katydid collection localities on Barro Colorado Island, with pie charts representing the plant families that were recovered from katydid stomachs by location and year. Each black dot represents a light capture location, with lights divided into three spatially and elevationally clustered zones. The inset pie charts represent the plant families that were sequenced in a given zone and year. “Conflict” indicates that different primer sets show different plant species identifications for a given individual katydid.

Successful plant sequences were recovered from the stomach contents of 71 insects representing 17 Neotropical forest katydid species. These katydid species consumed a wide variety of plant families, with each katydid species consuming multiple families of plants (Figure 1), and multiple katydid species consuming the same plant species. Comparing 2016 to 2017, katydids sampled from the same locations were often eating different plant families in different years (Figure 2).

**Figure 3.**
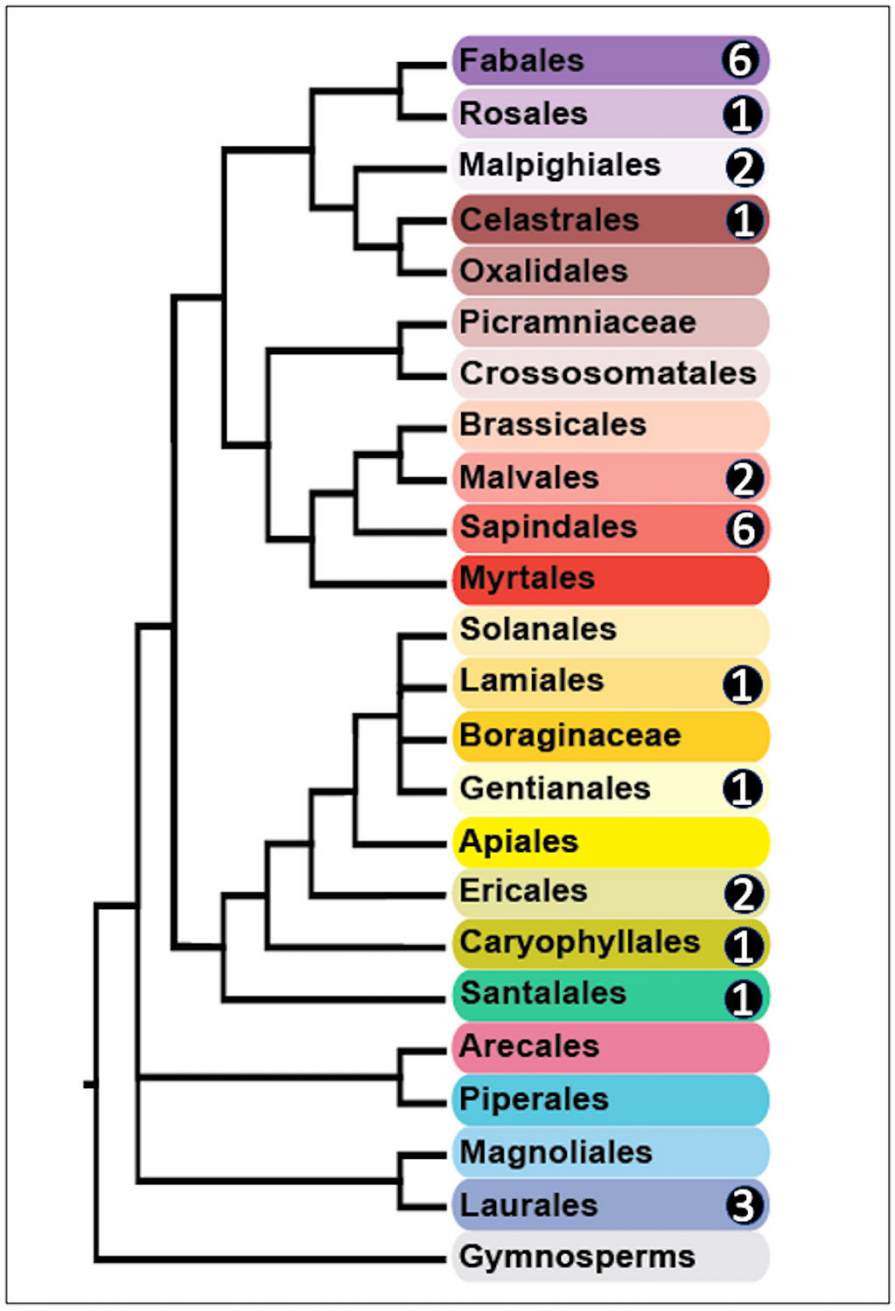
The phylogenetic distribution of plant species in the diets of katydids on BCI. The evolutionary relationships of the 23 orders of flowering plants found on BCI are represented in the branching diagram (modified from Figure 1 in [39]; see Figure S1 for a full representation of the species diversity of trees in the 50-ha forest dynamics plot). Circled numbers indicate the number of host plant species per order detected in the gut contents of katydids, as determined by DNA barcoding.

In total 27 species of host plants were determined from the gut contents of the sampled katydids by DNA barcodes (Table 1 and Table S3). These 27 species were distributed across 22 genera in 16 families and 12 orders and were phylogenetically spread across the 23 orders of flowering trees on Barro Colorado Island (Figure 3 and Figure S1). The largest numbers of species were found in the Fabales (six species), Sapindales (six species), and Laurales (three species). The remaining nine orders each had one or two species of host plants. Relatively few or no host species were detected in the speciose orders Gentianales, Rosales, Malpighiales, and Myrtales.

**Figure 4.**
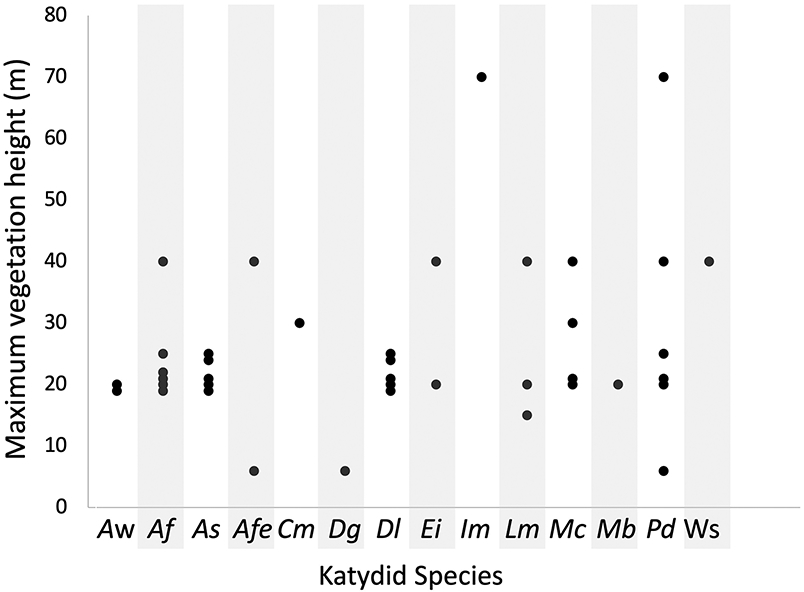
Maximum published growth height for the plants contained in the katydid diet, shown by katydid species. In the case of repeated plant height values, points are jittered slightly to show all data. Plants that could not be identified to species or genus level are not included due to variability of family-level maximum growth height. Katydid species abbreviations (*Aw: Anaulacomera* “wallace”; *Af: Anaulacomera furcata; As: Anaulacomera spatulata; Afe: Arota festae; Cm: Ceraia mytra; Dg: Docidocercus gigliotosi; Dl: Dolichocercus latipennis; Ei: Euceraia insignis; Im: Idiarthron major; Lm: Lamprophyllum micans; Mc: Microcentrum championi; Mb: Montezumina bradleyi; Pd: Phylloptera dimidiata;* Ws: Waxy sp.).

Most katydid species were consuming plant species that could grow into the canopy layer (Figure 4). However, some katydid species were foraging on plants that never grow higher than the understory.

## 4. Discussion

The results of our study do not support the hypothesis that Neotropical katydid species specialize their diet by host plant. By extracting plant DNA from the digestive systems of katydids, we were able to use DNA barcoding to identify which plant species and/or families were recently consumed. Individual katydids of the same species often had multiple and different plant families in their digestive systems, indicating that katydid species were not specializing on single host plants. In addition, the use of multiple primers sometimes recovered different plant species from the same katydid, providing evidence that katydids would feed on a diversity of plants even at short timescales. Consequently, dietary specialization on a specific host plant is not providing these species with a means of facilitating mate localization in the face of intense predation on signaling males and mate-searching females.

In contrast, the katydid species studied here were consuming a wide variety of plant families (16) and orders (12) comprising over half the orders of plants found on BCI. While many plant families and orders appeared in the katydid diet, some were particularly well-represented. Within the katydid samples that yielded a single identified plant order, 27% of the samples were Laurales and 23% were Fabales. No other plant family or order comprised more than 8% of the samples (Table 1). The abundance of Laurales and Fabales in the diet appears to be consistent with the relative abundance of stems of these plant orders on Barro Colorado Island, although species diversity in several other lineages, such as Gentianales, Myrtales, Rosales, and Malpighiales, is equally high or higher, suggesting that katydids may avoid some lineages of plants in favor of others [47-49]. Furthermore, it should be noted that the katydid specimens were collected primarily in January when certain trees may be in the young leaf stage and hence easier to digest than other species. If this is the case, then our results may in part be dependent on tree phenology. Further sampling at different times of the year in different seasons is certainly warranted.

The dietary composition of katydids provides one-directional information about the height at which katydids are foraging. Because even canopy emergent tree species start as saplings, the presence of canopy species in the katydid diet does not necessarily mean that the species was consumed in the canopy. In contrast, when katydids are eating shrubs and understory trees, it is strong evidence that the species is foraging low in the forest. While sample sizes are small for some katydid species, katydid species that are observed at ground level are in fact consuming understory vegetation (e.g., *Docidocercus gigliotosi* [22]) (Figure 4).

One notable finding was that many plant families were detected in only one year. In part, the year-to-year differences likely reflect the fact that there are many plant families and most were represented with relatively low frequency. However, the fact that some plant families are well-represented in one year and rare or absent in others suggests that there may be times when particular plant families or individual plants are especially palatable. For example, in a given sampling location on a given night, multiple katydids of multiple species had the same plant in their stomach (Table S3), perhaps reflecting a nearby feeding opportunity that attracted multiple species of katydid.

Transient feeding opportunities on particular plants would be consistent with what is known about the palatability and phenology of many tropical plants. Even though leaves may persist for several years, 25–70% of leaf damage occurs in the weeks when leaves are expanding [50,51]. In response to herbivore pressure, tropical plants have evolved a variety of strategies to minimize their window of vulnerability to herbivores [27]. Herbivore evasion strategies can include exceptionally rapid expansion of leaves, delayed greening, and synchronous flushing, strategies that minimize exposure to herbivory by compressing the window when leaves are maximally palatable [52,53].

The possibility of short-term feeding windows on specific plants is also supported by anecdotal field observations during this study, where katydids of multiple species were observed aggregated on a tree a few days before the tree produced substantial and obvious new growth (L. Symes, personal observation).

If katydids do aggregate to exploit transient feeding opportunities, co-localization on food sources might still facilitate mate finding, even for diet generalists. There are several avenues of investigation that could provide information on whether katydids aggregate to feed on plants during leafout. One strategy is to bait traps with plant volatiles such as benzyl nitrile, phenyl acetaldehyde, and/or 2-phenylethanol [54,55] to determine whether these chemical compounds are attractive to katydids, suggesting targeted feeding on vulnerable plants. A second strategy is to deploy long-term acoustic recorders and test for periods of time when a single location on the landscape has an elevated number of katydid calls, reflecting aggregation or one or more katydid species in an area for a short period of time. Understanding whether katydids aggregate on plants that are producing new leaves is important for understanding herbivore pressures on the forest vegetation, patterns of food availability for insect predators, and impacts of habitat patch size on insect populations and foraging effectiveness.

The results of DNA barcoding, when applied to Neotropical forest katydids, helps to define previously unseen connections between plants and herbivores, including connections that occur out of sight in the forest canopy. The generality of the katydid diet is more or less consistent with observations on other species in other locations, but underscores our lack of knowledge of how animals with rare short-range signals find each other in tropical forests. Our observations also suggest several avenues for future research.

## Supplementary Material

Supplemental materials

## Figures and Tables

**Table 1.** Plant species identified by DNA barcoding from the digestive tracts of Neotropical katydids.

Katydid Species	Plant Order	Plant Family	Plant Genus	Plant Species	Growth Habit
*Anaulacomera* “wallace”	Laurales (2*)	Lauraceae (2*)	Nectandra (2*)	lineata (2*)	Understory tree (10–25m)
*Anaulacomera furcata (v)*	*Ericales* *	*Sapotaceae* *	*Pouteria* *	*fossicola* *	Understory tree (10–25m)
	Fabales (2+1*)	Fabaceae (2+1*)	*Inga*	*sp*	
			*Swartzia* *	*simplex* *	Understory tree (10–25m)
			*Tachigali*	*versicolor*	Canopy tree (>25m)
	*Laurales* *	*Lauraceae* *	*Nectandra* *	*lineata* *	Understory tree (10–25m)
	*Rosales* *	*Cannabaceae* *	*Trema* *	*micrantha* *	Understory tree (10–25m)
	*Sapindales*	*Sapindaceae*	*Cupania*	*cinerea*	Understory tree (10–25m)
*Anaulacomera spatulata*	*Ericales*	*Lecythidaceae*	*Gustavia*	*superba*	Understory tree (10–25m)
	Fabales (1+2*)	Fabaceae (1+2*)	Inga (2*)	*goldmanii*	Understory tree (10–25m)
				*punctata*	Understory tree (10–25m)
			–	–	
	Laurales (3*)	Lauraceae (3*)	Nectandra (3*)	lineata (3*)	Understory tree (10–25m)
	*Sapindales*	*Sapindaceae*	*Cupania*	*rufescens*	Understory tree (10–25m)
*Arota festae*	*Fabales* *	Fabaceae*	*Inga* *	–	
	*Malpighiales* *	*Malpighiaceae* *	*Malpighia* *	*romeroana* *	Shrub (<6m)
	*Sapindales* *	*Anacardiaceae* *	*Anacardium* *	*excelsum* *	Canopy tree (>25m)
*Ceraia mytra*	*Lamiales*	*Bignoniaceae*	*Jacaranda*	*copaia*	Canopy tree (>25m)
*Docidocercus gigliotosi*	*Malpighiales*	*Malpighiaceae*	*Malpighia*	*romeroana*	Shrub (<6m)
*Dolichocercus latipennis*	Celastrales (1+1*)	Celastraceae (1+1*)	Maytenus (1+1*)	schippii (1+1*)	Understory tree (10–25m)
	Laurales (6+1*)	Lauraceae (6+1*)	Nectandra (5+1*)	lineata (5+1*)	Understory tree (10–25m)
			*Ocotea*	–	Understory tree (10–25m)
	*Malvales*	*Malvaceae*	–	–	
	*Santalales*	*Olacaceae*	*Heisteria*	*concinna*	Understory tree (10–25m)
	*Sapindales*	*Meliaceae*	*Guarea*	–	Understory tree (10–25m)
*Euceraia insignis*	Sapindales (2*)	*Anacardiaceae* *	*Anacardium* *	*excelsum* *	Canopy tree (>25m)
		*Sapindaceae* *	*Cupania* *	–	Understory tree (10–25m)
*Hyperphrona irregularis*	*Fabales* *	*Fabaceae* *	*Inga* *	–	
*Idiarthron major*	Fabales (2*)	Fabaceae (2*)	*Dipteryx* *	*oleifera*	Canopy tree (>25m)
			*Inga* *	–	
*Lamprophyllum micans*	*Gentianales*	*Rubiaceae*	*Chimarrhis*	*parviflora*	Understory tree (10–25m)
	Laurales (1+1*)	Lauraceae (1+1*)	Nectandra (1+1*)	lineata (1+1*)	Understory tree (10–25m)
	Sapindales (1+1*)	Anacardiaceae (1+1*)	Anacardium (1+1*)	excelsum (1+1*)	Canopy tree (>25m)
*Microcentrum* “polka”	*Fabales* *	*Fabaceae* *	*Inga* *	–	
*Microcentrum championi*	*Laurales*	*Lauraceae*	*Ocotea*	*puberula*	Understory tree (10–25m)
	*Malvales*	*Malvaceae*	*Luehea*	*seemannii*	Canopy tree (>25m)
	Sapindales (1+1*)	*Anacardiaceae* *	*Spondias* *	*radlkoferi* *	Canopy tree (>25m)
		*Sapindaceae*	*Cupania*	*cinerea*	Understory tree (10–25m)
*Montezumina bradleyi*	*Fabales (3)*	*Fabaceae (3)*	*Inga (3)*	–	
	*Laurales (3)*	*Lauraceae (3)*	*Nectandra (3)*	*lineata (3)*	Understory tree (10–25m)
*Phylloptera dimidiata*	*Caryophyllales*	*Nyctaginaceae*	*Guapira*	*standleyana*	Canopy tree (>25m)
	*Fabales* *	*Fabaceae* *	*Swartzia* *	*simplex* *	Understory tree (10–25m)
	Malpighiales (1+1*)	*Malpighiaceae*	*Malpighia*	*romeroana*	Shrub (<6m)
		*Phyllanthaceae* *	*Margaritaria* *	*nobilis* *	Understory tree (10–25m)
	Malvales (2+1*)	Malvaceae (2+1*)	Ceiba (2+1*)	pentandra (2+1*)	Canopy tree (>25m)
	*Sapindales* *	*Sapindaceae* *	*Cupania* *	*latifolia* *	Understory tree (10–25m)
“Waxy” *sp.*	*Fabales*	*Fabaceae*	*Inga*	*sp*	
	*Sapindales*	*Anacardiaceae*	*Anacardium*	*excelsum*	Canopy tree (>25m)

Plant species listed are supported by two or more primer sets. Plant species with * are supported by a single primer. The paranthetical number indicates the number of individual katydids associated with the identified plant.

## Data Availability

Sequence data for chloroplast DNA amplified from gut contents is available in the Supplementary Materials. Reference plant barcodes (accessions found in Table S2) can be found at https://www.ncbi.nlm.nih.gov/genbank/ (accessed on 10 August 2021).
