# Supplementary material for: Patterns of Herbivory in Neotropical Forest Katydids as Revealed by DNA Barcoding of Digestive Tract Contents
Source: Diversity (Basel). Author manuscript; Available in PMC 2022 Apr 1. (PMC8974511; doi:10.3390/d14020152)
Supplement: Supplemental materials [file NIHMS1784086-supplement-Supplemental_materials.zip › Table_S1.docx]

**Table S1**: Primers utilized for DNA barcode amplification

| **Amplicon** | **Primer** | **Direction** | **Sequence** |
| --- | --- | --- | --- |
| rbc | rbcLa_SI_For | Forward | 5'-ATGTCACCACAAACAGAGACTAAAGC-3' |
| rbc | rbcLa_SI_Rev | Reverse | 5'-GTAAAATCAAGTCCACCRCG-3' |
| psb | psbA3'f | Forward | 5'-GTTATGCATGAACGTAATGCTC-3' |
| psb | trnH | Reverse | 5'-CGCGCATGGTGGATTCACAATCC-3' |
| matK | matKfor_KIM3F | Forward | 5'-CGTACACAGTACTTTTGTGTTTACGAG-3' |
| matK | matKrev_KIM1R | Reverse | 5'-ACCCAGTCCATCTGAAATCTTGGTTC-3' |
