# Supplementary material for: Patterns of Herbivory in Neotropical Forest Katydids as Revealed by DNA Barcoding of Digestive Tract Contents
Source: Diversity (Basel). Author manuscript; Available in PMC 2022 Apr 1. (PMC8974511; doi:10.3390/d14020152)
Supplement: Supplemental materials [file NIHMS1784086-supplement-Supplemental_materials.zip › Table_S2.docx]

**Table S2**: List of accessions of DNA barcodes for BCI trees/shrubs/lianas used in BLAST searches

| **Accession** |
| --- |
| GQ981652 |
| GQ981653 |
| GQ981654 |
| GQ981655 |
| GQ981656 |
| GQ981657 |
| GQ981658 |
| GQ981659 |
| GQ981660 |
| GQ981661 |
| GQ981662 |
| GQ981663 |
| GQ981664 |
| GQ981665 |
| GQ981666 |
| GQ981667 |
| GQ981668 |
| GQ981669 |
| GQ981670 |
| GQ981671 |
| GQ981672 |
| GQ981673 |
| GQ981674 |
| GQ981675 |
| GQ981676 |
| GQ981677 |
| GQ981678 |
| GQ981679 |
| GQ981680 |
| GQ981681 |
| GQ981682 |
| GQ981683 |
| GQ981684 |
| GQ981685 |
| GQ981686 |
| GQ981687 |
| GQ981688 |
| GQ981689 |
| GQ981690 |
| GQ981691 |
| GQ981692 |
| GQ981693 |
| GQ981694 |
| GQ981695 |
| GQ981696 |
| GQ981697 |
| GQ981698 |
| GQ981699 |
| GQ981700 |
| GQ981701 |
| GQ981702 |
| GQ981703 |
| GQ981704 |
| GQ981705 |
| GQ981706 |
| GQ981707 |
| GQ981708 |
| GQ981709 |
| GQ981710 |
| GQ981711 |
| GQ981712 |
| GQ981713 |
| GQ981714 |
| GQ981715 |
| GQ981716 |
| GQ981717 |
| GQ981718 |
| GQ981719 |
| GQ981720 |
| GQ981721 |
| GQ981722 |
| GQ981723 |
| GQ981724 |
| GQ981725 |
| GQ981726 |
| GQ981727 |
| GQ981728 |
| GQ981729 |
| GQ981730 |
| GQ981731 |
| GQ981732 |
| GQ981733 |
| GQ981734 |
| GQ981735 |
| GQ981736 |
| GQ981737 |
| GQ981738 |
| GQ981739 |
| GQ981740 |
| GQ981741 |
| GQ981742 |
| GQ981743 |
| GQ981744 |
| GQ981745 |
| GQ981746 |
| GQ981747 |
| GQ981748 |
| GQ981749 |
| GQ981750 |
| GQ981751 |
| GQ981752 |
| GQ981753 |
| GQ981754 |
| GQ981755 |
| GQ981756 |
| GQ981757 |
| GQ981758 |
| GQ981759 |
| GQ981760 |
| GQ981761 |
| GQ981762 |
| GQ981763 |
| GQ981764 |
| GQ981765 |
| GQ981766 |
| GQ981767 |
| GQ981768 |
| GQ981769 |
| GQ981770 |
| GQ981771 |
| GQ981772 |
| GQ981773 |
| GQ981774 |
| GQ981775 |
| GQ981776 |
| GQ981777 |
| GQ981778 |
| GQ981779 |
| GQ981780 |
| GQ981781 |
| GQ981782 |
| GQ981783 |
| GQ981784 |
| GQ981785 |
| GQ981786 |
| GQ981787 |
| GQ981788 |
| GQ981789 |
| GQ981790 |
| GQ981791 |
| GQ981792 |
| GQ981793 |
| GQ981794 |
| GQ981795 |
| GQ981796 |
| GQ981797 |
| GQ981798 |
| GQ981799 |
| GQ981800 |
| GQ981801 |
| GQ981802 |
| GQ981803 |
| GQ981804 |
| GQ981805 |
| GQ981806 |
| GQ981807 |
| GQ981808 |
| GQ981809 |
| GQ981810 |
| GQ981811 |
| GQ981812 |
| GQ981813 |
| GQ981814 |
| GQ981815 |
| GQ981816 |
| GQ981817 |
| GQ981818 |
| GQ981819 |
| GQ981820 |
| GQ981821 |
| GQ981822 |
| GQ981823 |
| GQ981824 |
| GQ981825 |
| GQ981826 |
| GQ981827 |
| GQ981828 |
| GQ981829 |
| GQ981830 |
| GQ981831 |
| GQ981832 |
| GQ981833 |
| GQ981834 |
| GQ981835 |
| GQ981836 |
| GQ981837 |
| GQ981838 |
| GQ981839 |
| GQ981840 |
| GQ981841 |
| GQ981842 |
| GQ981843 |
| GQ981844 |
| GQ981845 |
| GQ981846 |
| GQ981847 |
| GQ981848 |
| GQ981849 |
| GQ981850 |
| GQ981851 |
| GQ981852 |
| GQ981853 |
| GQ981854 |
| GQ981855 |
| GQ981856 |
| GQ981857 |
| GQ981858 |
| GQ981859 |
| GQ981860 |
| GQ981862 |
| GQ981863 |
| GQ981864 |
| GQ981865 |
| GQ981866 |
| GQ981867 |
| GQ981868 |
| GQ981869 |
| GQ981870 |
| GQ981871 |
| GQ981872 |
| GQ981873 |
| GQ981874 |
| GQ981875 |
| GQ981876 |
| GQ981877 |
| GQ981878 |
| GQ981879 |
| GQ981880 |
| GQ981881 |
| GQ981882 |
| GQ981883 |
| GQ981884 |
| GQ981885 |
| GQ981886 |
| GQ981887 |
| GQ981888 |
| GQ981889 |
| GQ981890 |
| GQ981891 |
| GQ981892 |
| GQ981893 |
| GQ981894 |
| GQ981895 |
| GQ981896 |
| GQ981897 |
| GQ981898 |
| GQ981899 |
| GQ981900 |
| GQ981901 |
| GQ981902 |
| GQ981903 |
| GQ981904 |
| GQ981905 |
| GQ981906 |
| GQ981907 |
| GQ981908 |
| GQ981909 |
| GQ981910 |
| GQ981911 |
| GQ981912 |
| GQ981913 |
| GQ981914 |
| GQ981915 |
| GQ981916 |
| GQ981917 |
| GQ981918 |
| GQ981919 |
| GQ981920 |
| GQ981921 |
| GQ981922 |
| GQ981923 |
| GQ981924 |
| GQ981925 |
| GQ981926 |
| GQ981928 |
| GQ981929 |
| GQ981932 |
| GQ981933 |
| GQ981934 |
| GQ981937 |
| GQ981938 |
| GQ981939 |
| GQ981944 |
| GQ981946 |
| GQ981947 |
| GQ981948 |
| GQ981949 |
| GQ981950 |
| GQ981951 |
| GQ981958 |
| GQ981961 |
| GQ981963 |
| GQ981964 |
| GQ981965 |
| GQ981966 |
| GQ981969 |
| GQ981970 |
| GQ981973 |
| GQ981974 |
| GQ981978 |
| GQ981979 |
| GQ981980 |
| GQ981981 |
| GQ981982 |
| GQ981983 |
| GQ981988 |
| GQ981989 |
| GQ981990 |
| GQ981991 |
| GQ981992 |
| GQ981993 |
| GQ981995 |
| GQ981999 |
| GQ982001 |
| GQ982002 |
| GQ982003 |
| GQ982005 |
| GQ982007 |
| GQ982008 |
| GQ982009 |
| GQ982010 |
| GQ982011 |
| GQ982012 |
| GQ982016 |
| GQ982017 |
| GQ982022 |
| GQ982024 |
| GQ982028 |
| GQ982035 |
| GQ982040 |
| GQ982043 |
| GQ982045 |
| GQ982047 |
| GQ982049 |
| GQ982050 |
| GQ982053 |
| GQ982055 |
| GQ982056 |
| GQ982057 |
| GQ982060 |
| GQ982064 |
| GQ982065 |
| GQ982067 |
| GQ982068 |
| GQ982070 |
| GQ982074 |
| GQ982075 |
| GQ982079 |
| GQ982081 |
| GQ982083 |
| GQ982085 |
| GQ982086 |
| GQ982087 |
| GQ982088 |
| GQ982091 |
| GQ982092 |
| GQ982093 |
| GQ982094 |
| GQ982096 |
| GQ982097 |
| GQ982098 |
| GQ982101 |
| GQ982102 |
| GQ982103 |
| GQ982106 |
| GQ982107 |
| GQ982108 |
| GQ982109 |
| GQ982110 |
| GQ982111 |
| GQ982112 |
| GQ982116 |
| GQ982117 |
| GQ982121 |
| GQ982127 |
| GQ982128 |
| GQ982131 |
| GQ982133 |
| GQ982134 |
| GQ982135 |
| GQ982136 |
| GQ982137 |
| GQ982138 |
| GQ982139 |
| GQ982140 |
| GQ982141 |
| GQ982142 |
| GQ982143 |
| GQ982144 |
| GQ982145 |
| GQ982146 |
| GQ982147 |
| GQ982148 |
| GQ982149 |
| GQ982150 |
| GQ982151 |
| GQ982152 |
| GQ982153 |
| GQ982154 |
| GQ982155 |
| GQ982156 |
| GQ982157 |
| GQ982158 |
| GQ982159 |
| GQ982160 |
| GQ982161 |
| GQ982162 |
| GQ982163 |
| GQ982164 |
| GQ982165 |
| GQ982166 |
| GQ982167 |
| GQ982168 |
| GQ982169 |
| GQ982170 |
| GQ982171 |
| GQ982172 |
| GQ982173 |
| GQ982174 |
| GQ982175 |
| GQ982176 |
| GQ982177 |
| GQ982178 |
| GQ982179 |
| GQ982180 |
| GQ982181 |
| GQ982182 |
| GQ982183 |
| GQ982184 |
| GQ982185 |
| GQ982186 |
| GQ982187 |
| GQ982188 |
| GQ982189 |
| GQ982190 |
| GQ982191 |
| GQ982192 |
| GQ982193 |
| GQ982194 |
| GQ982195 |
| GQ982196 |
| GQ982197 |
| GQ982198 |
| GQ982199 |
| GQ982200 |
| GQ982201 |
| GQ982202 |
| GQ982203 |
| GQ982204 |
| GQ982205 |
| GQ982206 |
| GQ982207 |
| GQ982208 |
| GQ982209 |
| GQ982210 |
| GQ982211 |
| GQ982212 |
| GQ982213 |
| GQ982214 |
| GQ982215 |
| GQ982216 |
| GQ982217 |
| GQ982218 |
| GQ982219 |
| GQ982220 |
| GQ982221 |
| GQ982222 |
| GQ982223 |
| GQ982224 |
| GQ982225 |
| GQ982226 |
| GQ982227 |
| GQ982228 |
| GQ982229 |
| GQ982230 |
| GQ982231 |
| GQ982232 |
| GQ982233 |
| GQ982234 |
| GQ982235 |
| GQ982236 |
| GQ982237 |
| GQ982238 |
| GQ982239 |
| GQ982240 |
| GQ982241 |
| GQ982242 |
| GQ982243 |
| GQ982244 |
| GQ982245 |
| GQ982246 |
| GQ982247 |
| GQ982248 |
| GQ982249 |
| GQ982250 |
| GQ982251 |
| GQ982252 |
| GQ982253 |
| GQ982254 |
| GQ982255 |
| GQ982256 |
| GQ982257 |
| GQ982258 |
| GQ982259 |
| GQ982260 |
| GQ982261 |
| GQ982262 |
| GQ982263 |
| GQ982264 |
| GQ982265 |
| GQ982266 |
| GQ982267 |
| GQ982268 |
| GQ982269 |
| GQ982270 |
| GQ982271 |
| GQ982272 |
| GQ982273 |
| GQ982274 |
| GQ982275 |
| GQ982276 |
| GQ982277 |
| GQ982278 |
| GQ982279 |
| GQ982280 |
| GQ982281 |
| GQ982282 |
| GQ982283 |
| GQ982284 |
| GQ982285 |
| GQ982286 |
| GQ982287 |
| GQ982288 |
| GQ982289 |
| GQ982290 |
| GQ982291 |
| GQ982292 |
| GQ982293 |
| GQ982294 |
| GQ982295 |
| GQ982296 |
| GQ982297 |
| GQ982298 |
| GQ982299 |
| GQ982300 |
| GQ982301 |
| GQ982302 |
| GQ982303 |
| GQ982304 |
| GQ982305 |
| GQ982306 |
| GQ982307 |
| GQ982308 |
| GQ982309 |
| GQ982310 |
| GQ982311 |
| GQ982312 |
| GQ982313 |
| GQ982314 |
| GQ982315 |
| GQ982316 |
| GQ982317 |
| GQ982318 |
| GQ982319 |
| GQ982320 |
| GQ982321 |
| GQ982322 |
| GQ982323 |
| GQ982324 |
| GQ982325 |
| GQ982326 |
| GQ982327 |
| GQ982328 |
| GQ982329 |
| GQ982330 |
| GQ982331 |
| GQ982332 |
| GQ982333 |
| GQ982334 |
| GQ982335 |
| GQ982336 |
| GQ982337 |
| GQ982338 |
| GQ982339 |
| GQ982340 |
| GQ982341 |
| GQ982342 |
| GQ982343 |
| GQ982344 |
| GQ982345 |
| GQ982346 |
| GQ982347 |
| GQ982349 |
| GQ982350 |
| GQ982351 |
| GQ982352 |
| GQ982353 |
| GQ982354 |
| GQ982355 |
| GQ982356 |
| GQ982357 |
| GQ982358 |
| GQ982359 |
| GQ982360 |
| GQ982361 |
| GQ982362 |
| GQ982363 |
| GQ982364 |
| GQ982365 |
| GQ982366 |
| GQ982367 |
| GQ982368 |
| GQ982369 |
| GQ982370 |
| GQ982371 |
| GQ982372 |
| GQ982373 |
| GQ982374 |
| GQ982375 |
| GQ982376 |
| GQ982377 |
| GQ982378 |
| GQ982379 |
| GQ982380 |
| GQ982381 |
| GQ982382 |
| GQ982383 |
| GQ982384 |
| GQ982385 |
| GQ982386 |
| GQ982387 |
| GQ982388 |
| GQ982389 |
| GQ982390 |
| GQ982391 |
| GQ982392 |
| GQ982393 |
| GQ982394 |
| GQ982395 |
| GQ982396 |
| GQ982397 |
| GQ982398 |
| GQ982399 |
| GQ982400 |
| GQ982401 |
| GQ982403 |
| GQ982404 |
| GQ982405 |
| GQ982406 |
| GQ982407 |
| GQ982408 |
| GQ982409 |
| GQ982410 |
| GQ982411 |
| GQ982412 |
| KJ593753 |
| KJ593754 |
| KJ593755 |
| KJ593756 |
| KJ593757 |
| KJ593758 |
| KJ593759 |
| KJ593760 |
| KJ593761 |
| KJ593762 |
| KJ593763 |
| KJ593764 |
| KJ593765 |
| KJ593766 |
| KJ593767 |
| KJ593768 |
| KJ593769 |
| KJ593770 |
| KJ593771 |
| KJ593772 |
| KJ593773 |
| KJ593774 |
| KJ593775 |
| KJ593776 |
| KJ593777 |
| KJ593778 |
| KJ593779 |
| KJ593780 |
| KJ593781 |
| KJ593782 |
| KJ593783 |
| KJ593784 |
| KJ593785 |
| KJ593786 |
| KJ593787 |
| KJ593788 |
| KJ593789 |
| KJ593790 |
| KJ593791 |
| KJ593792 |
| KJ593793 |
| KJ593794 |
| KJ593795 |
| KJ593796 |
| KJ593797 |
| KJ593798 |
| KJ593799 |
| KJ593800 |
| KJ593801 |
| KJ593802 |
| KJ593803 |
| KJ593804 |
| KJ593805 |
| KJ593806 |
| KJ593807 |
| KJ593808 |
| KJ593809 |
| KJ593810 |
| KJ593811 |
| KJ593812 |
| KJ593813 |
| KJ593814 |
| KJ593815 |
| KJ593816 |
| KJ593817 |
| KJ593818 |
| KJ593819 |
| KJ593820 |
| KJ593821 |
| KJ593822 |
| KJ593823 |
| KJ593824 |
| KJ593825 |
| KJ593826 |
| KJ593827 |
| KJ593828 |
| KJ593829 |
| KJ593830 |
| KJ593831 |
| KJ593832 |
| KJ593833 |
| KJ593834 |
| KJ593835 |
| KJ593836 |
| KJ593837 |
| KJ593838 |
| KJ593839 |
| KJ593840 |
| KJ593841 |
| KJ593842 |
| KJ593843 |
| KJ593844 |
| KJ593845 |
| KJ593846 |
| KJ593847 |
| KJ593848 |
| KJ593849 |
| KJ593850 |
| KJ593851 |
| KJ593852 |
| KJ593853 |
| KJ593854 |
| KJ593855 |
| KJ593856 |
| KJ593857 |
| KJ593858 |
| KJ593859 |
| KJ593860 |
| KJ593861 |
| KJ593862 |
| KJ593863 |
| KJ593864 |
| KJ593865 |
| KJ593866 |
| KJ593867 |
| KJ593868 |
| KJ593869 |
| KJ593870 |
| KJ593871 |
| KJ593872 |
| KJ593873 |
| KJ593874 |
| KJ593875 |
| KJ593876 |
| KJ593877 |
| KJ593878 |
| KJ593879 |
| KJ593880 |
| KJ593881 |
| KJ593882 |
| KJ593883 |
| KJ593884 |
| KJ593885 |
| KJ593886 |
| KJ593887 |
| KJ593888 |
| KJ593889 |
| KJ593890 |
| KJ593891 |
| KJ593892 |
| KJ593893 |
| KJ593894 |
| KJ593895 |
| KJ593896 |
| KJ593897 |
| KJ593898 |
| KJ593899 |
| KJ593900 |
| KJ593901 |
| KJ593902 |
| KJ593903 |
| KJ593904 |
| KJ593905 |
| KJ593906 |
| KJ593907 |
| KJ593908 |
| KJ593909 |
| KJ593910 |
| KJ593911 |
| KJ593912 |
| KJ593913 |
| KJ593914 |
| KJ593915 |
| KJ593916 |
| KJ593917 |
| KJ593918 |
| KJ593919 |
| KJ593920 |
| KJ593921 |
| KJ593922 |
| KJ593923 |
| KJ593924 |
| KJ593925 |
| KJ593926 |
| KJ593927 |
| KJ593928 |
| KJ593929 |
| KJ593930 |
| KJ593931 |
| KJ593932 |
| KJ593933 |
| KJ593934 |
| KJ593935 |
| KJ593936 |
| KJ593937 |
| KJ593938 |
| KJ593939 |
| KJ593940 |
| KJ593941 |
| KJ593942 |
| KJ593943 |
| KJ593944 |
| KJ593945 |
| KJ593946 |
| KJ593947 |
| KJ593948 |
| KJ593949 |
| KJ593950 |
| KJ593951 |
| KJ593952 |
| KJ593953 |
| KJ593954 |
| KJ593955 |
| KJ593956 |
| KJ593957 |
| KJ593958 |
| KJ593959 |
| KJ593960 |
| KJ593961 |
| KJ593962 |
| KJ593963 |
| KJ593964 |
| KJ593965 |
| KJ593966 |
| KJ593967 |
| KJ593968 |
| KJ593969 |
| KJ593970 |
| KJ593971 |
| KJ593972 |
| KJ593973 |
| KJ593974 |
| KJ593975 |
| KJ593976 |
| KJ593977 |
| KJ593978 |
| KJ593979 |
| KJ593980 |
| KJ593981 |
| KJ593982 |
| KJ593983 |
| KJ593984 |
| KJ593985 |
| KJ593986 |
| KJ593987 |
| KJ593988 |
| KJ593989 |
| KJ593990 |
| KJ593991 |
| KJ593992 |
| KJ593993 |
| KJ593994 |
| KJ593995 |
| KJ593996 |
| KJ593997 |
| KJ593998 |
| KJ593999 |
| KJ594000 |
| KJ594001 |
| KJ594002 |
| KJ594003 |
| KJ594004 |
| KJ594005 |
| KJ594006 |
| KJ594007 |
| KJ594008 |
| KJ594009 |
| KJ594010 |
| KJ594011 |
| KJ594012 |
| KJ594013 |
| KJ594014 |
| KJ594015 |
| KJ594016 |
| KJ594017 |
| KJ594018 |
| KJ594019 |
| KJ594020 |
| KJ594021 |
| KJ594022 |
| KJ594023 |
| KJ594024 |
| KJ594025 |
| KJ594026 |
| KJ594027 |
| KJ594028 |
| KJ594029 |
| KJ594030 |
| KJ594031 |
| KJ594032 |
| KJ594033 |
| KJ594034 |
| KJ594035 |
| KJ594036 |
| KJ594037 |
| KJ594038 |
| KJ594039 |
| KJ594040 |
| KJ594041 |
| KJ594042 |
| KJ594043 |
| KJ594044 |
| KJ594045 |
| KJ594046 |
| KJ594047 |
| KJ594048 |
| KJ594049 |
| KJ594050 |
| KJ594051 |
| KJ594052 |
| KJ594053 |
| KJ594054 |
| KJ594055 |
| KJ594056 |
| KJ594057 |
| KJ594058 |
| KJ594059 |
| KJ594060 |
| KJ594061 |
| KJ594062 |
| KJ594063 |
| KJ594064 |
| KJ594065 |
| KJ594066 |
| KJ594067 |
| KJ594068 |
| KJ594069 |
| KJ594070 |
| KJ594071 |
| KJ594072 |
| KJ594073 |
| KJ594074 |
| KJ594075 |
| KJ594076 |
| KJ594077 |
| KJ594078 |
| KJ594079 |
| KJ594080 |
| KJ594084 |
| KJ594085 |
| KJ594086 |
| KJ594087 |
| KJ594088 |
| KJ594089 |
| KJ594090 |
| KJ594091 |
| KJ594092 |
| KJ594093 |
| KJ594094 |
| KJ594095 |
| KJ594096 |
| KJ594097 |
| KJ594098 |
| KJ594099 |
| KJ594100 |
| KJ594101 |
| KJ594102 |
| KJ594103 |
| KJ594104 |
| KJ594105 |
| KJ594106 |
| KJ594107 |
| KJ594108 |
| KJ594109 |
| KJ594110 |
| KJ594111 |
| KJ594112 |
| KJ594113 |
| KJ594114 |
| KJ594115 |
| KJ594116 |
| KJ594117 |
| KJ594118 |
| KJ594119 |
| KJ594120 |
| KJ594121 |
| KJ594122 |
| KJ594123 |
| KJ594124 |
| KJ594125 |
| KJ594126 |
| KJ594127 |
| KJ594128 |
| KJ594129 |
| KJ594130 |
| KJ594131 |
| KJ594132 |
| KJ594133 |
| KJ594134 |
| KJ594135 |
| KJ594136 |
| KJ594137 |
| KJ594138 |
| KJ594139 |
| KJ594140 |
| KJ594141 |
| KJ594142 |
| KJ594143 |
| KJ594144 |
| KJ594145 |
| KJ594146 |
| KJ594147 |
| KJ594148 |
| KJ594149 |
| KJ594150 |
| KJ594151 |
| KJ594152 |
| KJ594153 |
| KJ594154 |
| KJ594155 |
| KJ594156 |
| KJ594157 |
| KJ594158 |
| KJ594159 |
| KJ594160 |
| KJ594161 |
| KJ594162 |
| KJ594163 |
| KJ594164 |
| KJ594165 |
| KJ594166 |
| KJ594167 |
| KJ594168 |
| KJ594169 |
| KJ594170 |
| KJ594171 |
| KJ594172 |
| KJ594173 |
| KJ594174 |
| KJ594175 |
| KJ594176 |
| KJ594177 |
| KJ594178 |
| KJ594179 |
| KJ594180 |
| KJ594181 |
| KJ594182 |
| KJ594183 |
| KJ594184 |
| KJ594185 |
| KJ594186 |
| KJ594187 |
| KJ594188 |
| KJ594189 |
| KJ594190 |
| KJ594191 |
| KJ594192 |
| KJ594193 |
| KJ594194 |
| KJ594195 |
| KJ594196 |
| KJ594197 |
| KJ594198 |
| KJ594199 |
| KJ594200 |
| KJ594201 |
| KJ594202 |
| KJ594203 |
| KJ594204 |
| KJ594205 |
| KJ594206 |
| KJ594207 |
| KJ594208 |
| KJ594209 |
| KJ594210 |
| KJ594211 |
| KJ594212 |
| KJ594213 |
| KJ594214 |
| KJ594215 |
| KJ594216 |
| KJ594217 |
| KJ594218 |
| KJ594219 |
| KJ594220 |
| KJ594221 |
| KJ594222 |
| KJ594223 |
| KJ594224 |
| KJ594225 |
| KJ594226 |
| KJ594227 |
| KJ594228 |
| KJ594229 |
| KJ594230 |
| KJ594231 |
| KJ594232 |
| KJ594233 |
| KJ594234 |
| KJ594235 |
| KJ594236 |
| KJ594237 |
| KJ594238 |
| KJ594239 |
| KJ594240 |
| KJ594241 |
| KJ594242 |
| KJ594243 |
| KJ594244 |
| KJ594245 |
| KJ594246 |
| KJ594247 |
| KJ594248 |
| KJ594249 |
| KJ594250 |
| KJ594251 |
| KJ594252 |
| KJ594253 |
| KJ594254 |
| KJ594255 |
| KJ594256 |
| KJ594257 |
| KJ594258 |
| KJ594259 |
| KJ594260 |
| KJ594261 |
| KJ594262 |
| KJ594263 |
| KJ594264 |
| KJ594265 |
| KJ594266 |
| KJ594267 |
| KJ594268 |
| KJ594269 |
| KJ594270 |
| KJ594271 |
| KJ594272 |
| KJ594273 |
| KJ594274 |
| KJ594275 |
| KJ594276 |
| KJ594277 |
| KJ594278 |
| KJ594279 |
| KJ594280 |
| KJ594281 |
| KJ594282 |
| KJ594283 |
| KJ594284 |
| KJ594285 |
| KJ594286 |
| KJ594287 |
| KJ594288 |
| KJ594289 |
| KJ594290 |
| KJ594291 |
| KJ594292 |
| KJ594293 |
| KJ594294 |
| KJ594295 |
| KJ594296 |
| KJ594297 |
| KJ594298 |
| KJ594299 |
| KJ594300 |
| KJ594301 |
| KJ594302 |
| KJ594303 |
| KJ594304 |
| KJ594305 |
| KJ594306 |
| KJ594307 |
| KJ594308 |
| KJ594309 |
| KJ594310 |
| KJ594311 |
| KJ594312 |
| KJ594313 |
| KJ594314 |
| KJ594315 |
| KJ594316 |
| KJ594317 |
| KJ594318 |
| KJ594319 |
| KJ594320 |
| KJ594321 |
| KJ594322 |
| KJ594323 |
| KJ594324 |
| KJ594325 |
| KJ594326 |
| KJ594327 |
| KJ594328 |
| KJ594329 |
| KJ594330 |
| KJ594331 |
| KJ594332 |
| KJ594333 |
| KJ594334 |
| KJ594335 |
| KJ594336 |
| KJ594337 |
| KJ594338 |
| KJ594339 |
| KJ594340 |
| KJ594341 |
| KJ594342 |
| KJ594343 |
| KJ594344 |
| KJ594345 |
| KJ594346 |
| KJ594347 |
| KJ594348 |
| KJ594349 |
| KJ594350 |
| KJ594351 |
| KJ594352 |
| KJ594353 |
| KJ594354 |
| KJ594355 |
| KJ594356 |
| KJ594357 |
| KJ594358 |
| KJ594359 |
| KJ594360 |
| KJ594361 |
| KJ594362 |
| KJ594363 |
| KJ594364 |
| KJ594365 |
| KJ594366 |
| KJ594367 |
| KJ594368 |
| KJ594369 |
| KJ594370 |
| KJ594371 |
| KJ594372 |
| KJ594373 |
| KJ594374 |
| KJ594375 |
| KJ594376 |
| KJ594377 |
| KJ594378 |
| KJ594379 |
| KJ594380 |
| KJ594381 |
| KJ594382 |
| KJ594383 |
| KJ594384 |
| KJ594385 |
| KJ594386 |
| KJ594387 |
| KJ594388 |
| KJ594389 |
| KJ594390 |
| KJ594391 |
| KJ594392 |
| KJ594393 |
| KJ594394 |
| KJ594395 |
| KJ594396 |
| KJ594397 |
| KJ594398 |
| KJ594399 |
| KJ594400 |
| KJ594401 |
| KJ594402 |
| KJ594403 |
| KJ594404 |
| KJ594405 |
| KJ594406 |
| KJ594407 |
| KJ594408 |
| KJ594409 |
| KJ594410 |
| KJ594411 |
| KJ594412 |
| KJ594413 |
| KJ594414 |
| KJ594415 |
| KJ594416 |
| KJ594417 |
| KJ594418 |
| KJ594419 |
| KJ594420 |
| KJ594421 |
| KJ594422 |
| KJ594423 |
| KJ594424 |
| KJ594425 |
| KJ594426 |
| KJ594427 |
| KJ594428 |
| KJ594429 |
| KJ594430 |
| KJ594431 |
| KJ594432 |
| KJ594433 |
| KJ594434 |
| KJ594435 |
| KJ594436 |
| KJ594437 |
| KJ594438 |
| KJ594439 |
| KJ594440 |
| KJ594441 |
| KJ594442 |
| KJ594443 |
| KJ594444 |
| KJ594445 |
| KJ594446 |
| KJ594447 |
| KJ594448 |
| KJ594449 |
| KJ594450 |
| KJ594451 |
| KJ594452 |
| KJ594453 |
| KJ594454 |
| KJ594455 |
| KJ594456 |
| KJ594457 |
| KJ594458 |
| KJ594459 |
| KJ594460 |
| KJ594461 |
| KJ594462 |
| KJ594463 |
| KJ594464 |
| KJ594465 |
| KJ594466 |
| KJ594467 |
| KJ594468 |
| KJ594469 |
| KJ594470 |
| KJ594471 |
| KJ594472 |
| KJ594473 |
| KJ594474 |
| KJ594475 |
| KJ594476 |
| KJ594477 |
| KJ594478 |
| KJ594479 |
| KJ594480 |
| KJ594481 |
| KJ594482 |
| KJ594483 |
| KJ594484 |
| KJ594485 |
| KJ594486 |
| KJ594487 |
| KJ594488 |
| KJ594489 |
| KJ594490 |
| KJ594491 |
| KJ594492 |
| KJ594493 |
| KJ594494 |
| KJ594495 |
| KJ594496 |
| KJ594497 |
| KJ594498 |
| KJ594499 |
| KJ594500 |
| KJ594501 |
| KJ594502 |
| KJ594503 |
| KJ594504 |
| KJ594505 |
| KJ594506 |
| KJ594507 |
| KJ594508 |
| KJ594509 |
| KJ594510 |
| KJ594511 |
| KJ594512 |
| KJ594513 |
| KJ594514 |
| KJ594515 |
| KJ594516 |
| KJ594517 |
| KJ594518 |
| KJ594519 |
| KJ594520 |
| KJ594521 |
| KJ594522 |
| KJ594523 |
| KJ594524 |
| KJ594525 |
| KJ594526 |
| KJ594527 |
| KJ594528 |
| KJ594529 |
| KJ594530 |
| KJ594531 |
| KJ594532 |
| KJ594533 |
| KJ594534 |
| KJ594535 |
| KJ594536 |
| KJ594537 |
| KJ594538 |
| KJ594539 |
| KJ594540 |
| KJ594541 |
| KJ594542 |
| KJ594543 |
| KJ594544 |
| KJ594545 |
| KJ594546 |
| KJ594547 |
| KJ594548 |
| KJ594549 |
| KJ594550 |
| KJ594551 |
| KJ594552 |
| KJ594553 |
| KJ594554 |
| KJ594555 |
| KJ594556 |
| KJ594557 |
| KJ594558 |
| KJ594559 |
| KJ594560 |
| MZ478258 |
| MZ478259 |
| MZ478260 |
| MZ478261 |
| MZ478262 |
| MZ478263 |
| MZ478264 |
| MZ478265 |
| MZ478266 |
| MZ478267 |
| MZ478268 |
| MZ478269 |
| MZ478270 |
| MZ478271 |
| MZ478272 |
| MZ478273 |
| MZ478274 |
| MZ478275 |
| MZ478276 |
| MZ478277 |
| MZ478278 |
| MZ478279 |
| MZ478280 |
| MZ478281 |
| MZ478282 |
| MZ478283 |
| MZ478284 |
| MZ478285 |
| MZ478286 |
| MZ478287 |
| MZ478288 |
| MZ478289 |
| MZ478290 |
| MZ478291 |
| MZ478292 |
| MZ478293 |
| MZ478294 |
| MZ478295 |
| MZ478296 |
| MZ478297 |
| MZ478298 |
| MZ478299 |
| MZ478300 |
| MZ478301 |
| MZ478302 |
| MZ478303 |
| MZ478304 |
| MZ478305 |
| MZ478306 |
| MZ478307 |
| MZ478308 |
| MZ478309 |
| MZ478310 |
| MZ478311 |
| MZ478312 |
| MZ478313 |
| MZ478314 |
| MZ478315 |
| MZ478316 |
| MZ478317 |
| MZ478318 |
| MZ478319 |
| MZ478320 |
| MZ478321 |
| MZ478322 |
| MZ478323 |
| MZ478324 |
| MZ478325 |
| MZ478326 |
| MZ478327 |
| MZ478328 |
| MZ478329 |
| MZ478330 |
| MZ478331 |
| MZ478332 |
| MZ478333 |
| MZ478334 |
| MZ478335 |
| MZ478336 |
| MZ478337 |
| MZ478338 |
| MZ478339 |
| MZ478340 |
| MZ478341 |
| MZ478342 |
| MZ478343 |
| MZ478344 |
| MZ478345 |
| MZ478346 |
| MZ478347 |
| MZ478348 |
| MZ478349 |
| MZ478350 |
| MZ478351 |
| MZ478352 |
| MZ478353 |
| MZ478354 |
| MZ478355 |
| MZ478356 |
| MZ478357 |
| MZ478358 |
| MZ478359 |
| MZ478360 |
| MZ478361 |
| MZ478362 |
| MZ478363 |
| MZ478364 |
| MZ478365 |
| MZ478366 |
| MZ478367 |
| MZ478368 |
| MZ478369 |
| MZ478370 |
| MZ478371 |
| MZ478372 |
| MZ478373 |
| MZ478374 |
| MZ478375 |
| MZ478376 |
| MZ478377 |
| MZ478378 |
| MZ478379 |
| MZ478380 |
| MZ478381 |
| MZ478382 |
| MZ478383 |
| MZ478384 |
| MZ478385 |
| MZ478386 |
| MZ478387 |
| MZ478388 |
| MZ478389 |
| MZ478390 |
| MZ478391 |
| MZ478392 |
| MZ478393 |
| MZ478394 |
| MZ478395 |
| MZ478396 |
| MZ478397 |
| MZ478398 |
| MZ478399 |
| MZ478400 |
| MZ478401 |
| MZ478402 |
| MZ478403 |
| MZ478404 |
| MZ478405 |
| MZ478406 |
| MZ478407 |
| MZ478408 |
| MZ478410 |
| MZ478411 |
| MZ478412 |
| MZ478413 |
| MZ478414 |
| MZ478415 |
| MZ478416 |
| MZ478417 |
| MZ478418 |
| MZ478419 |
| MZ478420 |
| MZ478421 |
| MZ478422 |
| MZ478423 |
| MZ478424 |
| MZ478425 |
| MZ478426 |
| MZ478427 |
| MZ478428 |
| MZ478429 |
| MZ478430 |
| MZ478431 |
| MZ478432 |
| MZ478433 |
| MZ478434 |
| MZ478435 |
| MZ478436 |
| MZ478437 |
| MZ478438 |
| MZ478439 |
| MZ478440 |
| MZ478441 |
| MZ478442 |
| MZ478443 |
| MZ478444 |
| MZ478445 |
| MZ478446 |
| MZ478447 |
| MZ478448 |
| MZ478449 |
| MZ478450 |
| MZ478451 |
| MZ478452 |
| MZ478453 |
| MZ478454 |
| MZ478455 |
| MZ478456 |
| MZ478457 |
| MZ478458 |
| MZ478459 |
| MZ478460 |
| MZ478461 |
| MZ478462 |
| MZ478463 |
| MZ478464 |
| MZ478465 |
| MZ478466 |
| MZ478467 |
| MZ478468 |
| MZ478469 |
| MZ478470 |
| MZ478471 |
| MZ478472 |
| MZ478473 |
| MZ478474 |
| MZ478475 |
| MZ478476 |
| MZ478477 |
| MZ478478 |
| MZ478479 |
| MZ478480 |
| MZ478481 |
| MZ478482 |
| MZ478483 |
| MZ478484 |
| MZ478485 |
| MZ478486 |
| MZ478487 |
| MZ478488 |
| MZ478489 |
| MZ478490 |
| MZ478491 |
| MZ478492 |
| MZ478494 |
| MZ478495 |
| MZ478496 |
| MZ478497 |
| MZ478498 |
| MZ478499 |
| MZ478500 |
| MZ478501 |
| MZ478502 |
| MZ478503 |
| MZ478504 |
| MZ478505 |
| MZ478506 |
| MZ478507 |
| MZ478508 |
| MZ478509 |
| MZ478510 |
| MZ478511 |
| MZ478512 |
| MZ478513 |
| MZ478514 |
| MZ478515 |
| MZ478516 |
| MZ478518 |
| MZ478519 |
| MZ478521 |
| MZ478522 |
| MZ478523 |
| MZ478524 |
| MZ478525 |
| MZ478526 |
| MZ478527 |
| MZ478528 |
| MZ478529 |
| MZ478530 |
| MZ478531 |
| MZ478532 |
| MZ478533 |
| MZ478534 |
| MZ478535 |
| MZ478536 |
| MZ478537 |
| MZ478538 |
| MZ478539 |
| MZ478540 |
| MZ478541 |
| MZ478542 |
| MZ478543 |
| MZ478544 |
| MZ478545 |
| MZ478546 |
| MZ478547 |
| MZ478548 |
| MZ478549 |
| MZ478550 |
| MZ478551 |
| MZ478552 |
| MZ478553 |
| MZ478554 |
| MZ478555 |
| MZ478556 |
| MZ478557 |
| MZ478558 |
| MZ478559 |
| MZ478560 |
| MZ478561 |
| MZ478562 |
| MZ478563 |
| MZ478564 |
| MZ478565 |
| MZ478566 |
| MZ478567 |
| MZ478568 |
| MZ478569 |
| MZ478570 |
| MZ478571 |
| MZ478572 |
| MZ478573 |
| MZ478574 |
| MZ478575 |
| MZ478576 |
| MZ478577 |
| MZ478578 |
| MZ478579 |
| MZ478580 |
| MZ478581 |
| MZ478582 |
| MZ478583 |
| MZ478584 |
| MZ478585 |
| MZ478586 |
| MZ478587 |
| MZ478588 |
| MZ478589 |
| MZ478590 |
| MZ478591 |
| MZ478592 |
| MZ478593 |
| MZ478594 |
| MZ478595 |
| MZ478596 |
| MZ478597 |
| MZ478598 |
| MZ478599 |
| MZ478600 |
| MZ478601 |
| MZ478602 |
| MZ478603 |
| MZ478604 |
| MZ478605 |
| MZ478606 |
| MZ478607 |
| MZ478608 |
| MZ478609 |
| MZ493695 |
| MZ493696 |
| MZ493697 |
| MZ493698 |
| MZ493699 |
| MZ493700 |
| MZ493701 |
| MZ493702 |
| MZ493703 |
| MZ493704 |
| MZ493705 |
| MZ493706 |
| MZ493707 |
| MZ493708 |
| MZ493709 |
| MZ493710 |
| MZ493711 |
| MZ493712 |
| MZ493713 |
| MZ493714 |
| MZ493715 |
| MZ493716 |
| MZ493717 |
| MZ493718 |
| MZ493719 |
| MZ493720 |
| MZ493721 |
| MZ493722 |
| MZ493723 |
| MZ493724 |
| MZ493725 |
| MZ493726 |
| MZ493727 |
| MZ493728 |
| MZ493729 |
| MZ493730 |
| MZ493731 |
| MZ493732 |
| MZ493733 |
| MZ493734 |
| MZ493735 |
| MZ493736 |
| MZ493737 |
| MZ493738 |
| MZ493739 |
| MZ493740 |
| MZ493741 |
| MZ493742 |
| MZ493743 |
| MZ493744 |
| MZ493745 |
| MZ493746 |
| MZ493747 |
| MZ493748 |
| MZ493749 |
| MZ493750 |
| MZ493751 |
| MZ493752 |
| MZ493753 |
| MZ493754 |
| MZ493755 |
| MZ493756 |
| MZ493757 |
| MZ493758 |
| MZ493759 |
| MZ493760 |
| MZ493761 |
| MZ493762 |
| MZ493763 |
| MZ493764 |
| MZ493765 |
| MZ493766 |
| MZ493767 |
| MZ493768 |
| MZ493769 |
| MZ493770 |
| MZ493771 |
| MZ493772 |
| MZ493773 |
| MZ493774 |
| MZ493775 |
| MZ493776 |
| MZ493777 |
| MZ493778 |
| MZ493779 |
| MZ493780 |
| MZ493781 |
| MZ493782 |
| MZ493783 |
| MZ493784 |
| MZ493785 |
| MZ493786 |
| MZ493787 |
| MZ493788 |
| MZ493789 |
| MZ493790 |
| MZ493791 |
| MZ493792 |
| MZ493793 |
| MZ493794 |
| MZ493795 |
| MZ493796 |
| MZ493797 |
| MZ493798 |
| MZ493799 |
| MZ493800 |
| MZ493801 |
| MZ493802 |
| MZ493803 |
| MZ493804 |
| MZ493805 |
| MZ493806 |
| MZ493807 |
| MZ493808 |
| MZ493809 |
| MZ493810 |
| MZ493811 |
| MZ493812 |
| MZ493813 |
| MZ493814 |
| MZ493815 |
| MZ493816 |
| MZ493817 |
| MZ493818 |
| MZ493819 |
| MZ493820 |
| MZ493821 |
| MZ493822 |
| MZ493823 |
| MZ493824 |
| MZ493825 |
| MZ493826 |
| MZ493827 |
| MZ493828 |
| MZ493829 |
| MZ493830 |
| MZ493831 |
| MZ493832 |
| MZ493833 |
| MZ493834 |
| MZ493835 |
| MZ493836 |
| MZ493837 |
| MZ493838 |
| MZ493839 |
| MZ493840 |
| MZ493841 |
| MZ493842 |
| MZ493843 |
| MZ493844 |
| MZ493845 |
| MZ493846 |
| MZ493847 |
| MZ493848 |
| MZ493849 |
| MZ493850 |
| MZ493851 |
| MZ493852 |
| MZ493853 |
| MZ493854 |
| MZ493855 |
| MZ493856 |
| MZ493857 |
| MZ493858 |
| MZ493859 |
| MZ493860 |
| MZ493861 |
| MZ493862 |
| MZ493863 |
| MZ493864 |
| MZ493865 |
| MZ493866 |
| MZ493867 |
| MZ493868 |
| MZ493869 |
| MZ493870 |
| MZ493871 |
| MZ493872 |
| MZ493873 |
| MZ493874 |
| MZ493875 |
| MZ493876 |
| MZ493877 |
| MZ493878 |
| MZ493879 |
| MZ493880 |
| MZ493881 |
| MZ493882 |
| MZ493883 |
| MZ493884 |
| MZ493885 |
| MZ493886 |
| MZ493887 |
| MZ493888 |
| MZ493889 |
| MZ493890 |
| MZ493891 |
| MZ493892 |
| MZ493893 |
