# Supplementary material for: Patterns of Herbivory in Neotropical Forest Katydids as Revealed by DNA Barcoding of Digestive Tract Contents
Source: Diversity (Basel). Author manuscript; Available in PMC 2022 Apr 1. (PMC8974511; doi:10.3390/d14020152)
Supplement: Supplemental materials [file NIHMS1784086-supplement-Supplemental_materials.zip › Table_S3.docx]

**Table S3**: List of katydid samples, plant BLAST result, and collection metadata

| **Insect**  **ID** | **Insect**  **species** | **Plant**  **order** | **Plant**  **family** | **Plant**  **genus** | **Plant**  **species** | **Primers**  **supporting** | **Date** | **Time** | **Sex** | **Location** |
| --- | --- | --- | --- | --- | --- | --- | --- | --- | --- | --- |
| 4027 | *Lamprophyllum micans* | Gentianales | Rubiaceae | Chimarrhis | parviflora | rbc, psb | 20160102 | 2146 | m | VC |
| 4038 | *Dolichocercus latipennis* | Sapindales | Meliaceae | Guarea sp. |  | rbc, psb | 20160102 | 2059 | m | DH |
| 4022 | *Montezumina bradleyi* | Laurales | Lauraceae | Nectandra | lineata | rbc, psb | 20160103 | 456 | m | VC |
| 4002 | *Phylloptera dimidiata* | Malvales | Malvaceae | Ceiba | pentandra | rbc, matk | 20160103 | 515 | m | GH |
| 4007 | *Phylloptera dimidiata* | Malvales | Malvaceae | Ceiba | pentandra | rbc | 20160103 | 502 | f | VC |
| 4006 | *Phylloptera dimidiata* |  | conflict | conflict |  | rbc, psb | 20160103 | 2345 | m | F |
| 4014 | *Phylloptera dimidiata* | Caryophyllales | Nyctaginaceae | Guapira | standleyana | rbc, psb | 20160104 | 541 | m | IT |
| 4034 | *Dolichocercus latipennis* | Celastrales | Celastraceae | Maytenus | schippii | rbc, psb | 20160104 | 620 | m | CN |
| 4003 | *Arota festae* | Fabales | Fabaceae | Inga | sp | rbc | 20160104 | 51 | m | GH |
| 4005 | *Hyperphrona irregularis* | Fabales | Fabaceae | Inga |  | rbc | 20160104 | 31 | f | HH |
| 4004 | "Waxy" sp. | Fabales | Fabaceae | Inga | sp | rbc, psb | 20160104 | 41 | m | GH |
| 4045 | *Dolichocercus latipennis* | Malvales | Malvaceae | family level only |  | rbc, psb | 20160104 | 602 | m | VC |
| 4056 | *Idiarthron major* | Fabales | Fabaceae | Dipteryx | oleifera | rbc | 20160105 | 2326 | m | DH |
| 4058 | *Idiarthron major* | Fabales | Fabaceae | Inga |  | rbc | 20160105 | 2318 | m | F |
| 4040 | *Arota festae* | Malpighiales | Malpighiaceae | Malpighia | romeroana | rbc | 20160106 | 500 | m | G |
| 4053 | *Dolichocercus latipennis* | Celastrales | Celastraceae | Maytenus | schippii | rbc | 20160107 | 520 | f | DH |
| 4054 | *Dolichocercus latipennis* | Laurales | Lauraceae | Nectandra | lineata | rbc, psb | 20160107 | 530 | m | VC |
| 4055 | *Dolichocercus latipennis* | Laurales | Lauraceae | Nectandra | lineata | rbc, psb | 20160107 | 558 | m | VC |
| 4083 | *Euceraia insignis* |  | conflict | conflict |  | rbc, psb | 20160107 | 547 | m | IT |
| 4061 | *Dolichocercus latipennis* | Laurales | Lauraceae | Nectandra | lineata | rbc, psb | 20160108 | 532 | m | VC |
| 4086 | *Dolichocercus latipennis* | Laurales | Lauraceae | Nectandra | lineata | rbc, psb | 20160129 | 531 | m | VC |
| 4080 | *Phylloptera dimidiata* | Malvales | Malvaceae | Ceiba | pentandra | rbc, matk | 20160129 | 59 | f | VC |
| 4068 | "Waxy" sp. | Sapindales | Anacardiaceae | Anacardium | excelsum | rbc, matk | 20160130 | 528 | m | DH |
| 4087 | *Dolichocercus latipennis* | Laurales | Lauraceae | Nectandra | lineata | rbc, psb | 20160202 | 2247 | f | VC |
| 4073 | *Lamprophyllum micans* | Sapindales | Anacardiaceae | Anacardium | excelsum | rbc, matk | 20160202 | 24 | m | DH |
| 4067 | *Lamprophyllum micans* | Laurales | Lauraceae | Nectandra | lineata | rbc, psb | 20160205 | 2200 | m | Romer Lab |
| 4065 | *Lamprophyllum micans* |  | conflict | conflict |  | rbc, psb | 20160206 | 100 | m | F |
| 4081 | *Phylloptera dimidiata* | Malpighiales | Phyllanthaceae | Margaritaria | nobilis | rbc | 20160207 | 1 | m | GH |
| 4090 | *Phylloptera dimidiata* | Fabales | Fabaceae | Swartzia | simplex | rbc | 20160209 | 2308 | m | DH |
| 1 | *Anaulacomera furcata* | Laurales | Lauraceae | Nectandra | lineata | rbc | 20161229 | 1835 | f | CN |
| 32 | *Dolichocercus latipennis* | Laurales | Lauraceae | Nectandra | lineata | rbc | 20161230 | 2000 | f | Laundry |
| 79 | *Montezumina bradleyi* | Laurales | Lauraceae | Nectandra | lineata | rbc, psb | 20161230 | 1400 | m | VC |
| 90 | *Montezumina bradleyi* | Fabales | Fabaceae | Inga |  | rbc, psb, matk | 20161231 | 431 | m | VC |
| 91 | *Docidocercus gigliotosi* | Malpighiales | Malpighiaceae | Malpighia | romeroana | rbc, psb | 20161231 | 530 | m | C |
| 82 | *Dolichocercus latipennis* | Santalales | Olacaceae | Heisteria | concinna | rbc, matk | 20161231 | 530 | m | Laundry |
| 81 | *Dolichocercus latipennis* |  | conflict | conflict |  | rbc, psb | 20161231 | 535 | m | G |
| 229 | *Anaulacomera spatulata* | Laurales | Lauraceae | Nectandra | lineata | rbc | 20170100 | NA | m | Unk |
| 310 | *Anaulacomera furcata* | Fabales | Fabaceae | Swartzia | simplex | rbc | 20170102 | 2256 | f | NL |
| 281 | *Montezumina bradleyi* | Laurales | Lauraceae | Nectandra | lineata | rbc, psb | 20170102 | 2359 | f | HH |
| 324 | *Anaulacomera spatulata* | Ericales | Lecythidaceae | Gustavia | superba | rbc, psb | 20170103 | 554 | m | E |
| 228 | *Anaulacomera spatulata* | Fabales | Fabaceae | family level only |  | rbc, psb | 20170103 | 6 | f | DH |
| 265 | *Lamprophyllum micans* | Laurales | Lauraceae | Nectandra | lineata | rbc | 20170103 | 2357 | m | DH |
| 284 | *Anaulacomera furcata* | Rosales | Cannabaceae | Trema | micrantha | rbc | 20170103 | 18 | f | DH |
| 270 | *Anaulacomera "wallace"* | Laurales | Lauraceae | Nectandra | lineata | rbc | 20170104 | 32 | f | CTFS |
| 316 | *Anaulacomera "wallace"* | Laurales | Lauraceae | Nectandra | lineata | rbc | 20170104 | 30 | f | VC |
| 226 | *Anaulacomera spatulata* | Laurales | Lauraceae | Nectandra | lineata | rbc | 20170104 | 2313 | m | Laundry |
| 184 | *Anaulacomera spatulata* | Sapindales | Sapindaceae | Cupania | rufescens | rbc, psb | 20170104 | 2309 | m | NL |
| 306 | *Dolichocercus latipennis* |  | conflict | conflict |  | rbc, psb, matk | 20170104 | 30 | m | VC |
| 217 | *Anaulacomera furcata* | Fabales | Fabaceae | Inga | mixed | rbc, psb | 20170105 | 2346 | f | VC |
| 183 | *Anaulacomera spatulata* | Laurales | Lauraceae | Nectandra | lineata | rbc | 20170105 | 6 | m | VC |
| 180 | *Phylloptera dimidiata* |  | conflict | conflict |  | rbc, psb, matk | 20170105 | 3 | m | VC |
| 317 | *Dolichocercus latipennis* | Laurales | Lauraceae | Ocotea | sp | rbc, psb, matk | 20170106 | 502 | m | VC |
| 368 | *Montezumina bradleyi* | Fabales | Fabaceae | Inga | sp | rbc, psb, matk | 20170109 | 845 | f | VC |
| 375 | *Montezumina bradleyi* | Fabales | Fabaceae | Inga | sp | rbc, psb | 20170110 | 513 | f | VC |
| 377 | *Microcentrum championi* | Laurales | Lauraceae | Ocotea | puberula | rbc, psb | 20170110 | 559 | m | NL |
| 1594 | *Anaulacomera furcata* | Fabales | Fabaceae | Tachigali | versicolor | rbc, psb | 20170118 | 46 | f | DH |
| 1587 | *Phylloptera dimidiata* | Malpighiales | Malpighiaceae | Malpighia | romeroana | rbc, psb | 20170120 | 524 | f | IT |
| 1513 | *Anaulacomera spatulata* | Fabales | Fabaceae | Inga | punctata | psb | 20170122 | 2355 | f | E |
| 1484 | *Anaulacomera furcata* | Ericales | Sapotaceae | Pouteria | fossicola | rbc | 20170124 | 2343 | f | VC |
| 1528 | *Microcentrum "polka"* | Fabales | Fabaceae | Inga |  | rbc | 20170124 | 600 | m | F |
| 1604 | *Arota festae* | Sapindales | Anacardiaceae | Anacardium | excelsum | rbc | 20170124 | 40 | f | DH |
| 1575 | *Anaulacomera furcata* | Sapindales | Sapindaceae | Cupania | cinerea | rbc, psb | 20170124 | 140 | f | IT |
| 1567 | *Microcentrum championi* | Sapindales | Sapindaceae | Cupania | cinerea | rbc, psb, matk | 20170124 | 105 | f | DH |
| 1553 | *Microcentrum championi* | Sapindales | Anacardiaceae | Spondias | radlkoferi | rbc | 20170125 | 106 | m | SL |
| 1423 | *Microcentrum championi* | Malvales | Malvaceae | Luehea | seemannii | rbc, psb, matk | 20170206 | 452 | m | B |
| 1481 | *Anaulacomera spatulata* | Fabales | Fabaceae | Inga | goldmanii | rbc | 20170215 | 2338 | m | VC |
| 1243 | *Lamprophyllum micans* | Sapindales | Anacardiaceae | Anacardium | excelsum | rbc | 20170224 | 508 | m | CTFS |
| 1263 | *Euceraia insignis* | Sapindales | Anacardiaceae | Anacardium | excelsum | rbc | 20170226 | 537 | m | DH |
| 1251 | *Euceraia insignis* | Sapindales | Sapindaceae | Cupania | sp | psb | 20170301 | 502 | m | VC |
| 1407 | *Phylloptera dimidiata* | Sapindales | Sapindaceae | Cupania | latifolia | psb | 20170301 | 454 | f | CN |
| 1318 | *Ceraia mytra* | Lamiales | Bignoniaceae | Jacaranda | copaia | rbc, psb | 20170302 | 551 | f | VC |
