# Supplementary figures and images for: Patterns of Herbivory in Neotropical Forest Katydids as Revealed by DNA Barcoding of Digestive Tract Contents
Source: Diversity (Basel). Author manuscript; Available in PMC 2022 Apr 1. (PMC8974511; doi:10.3390/d14020152)

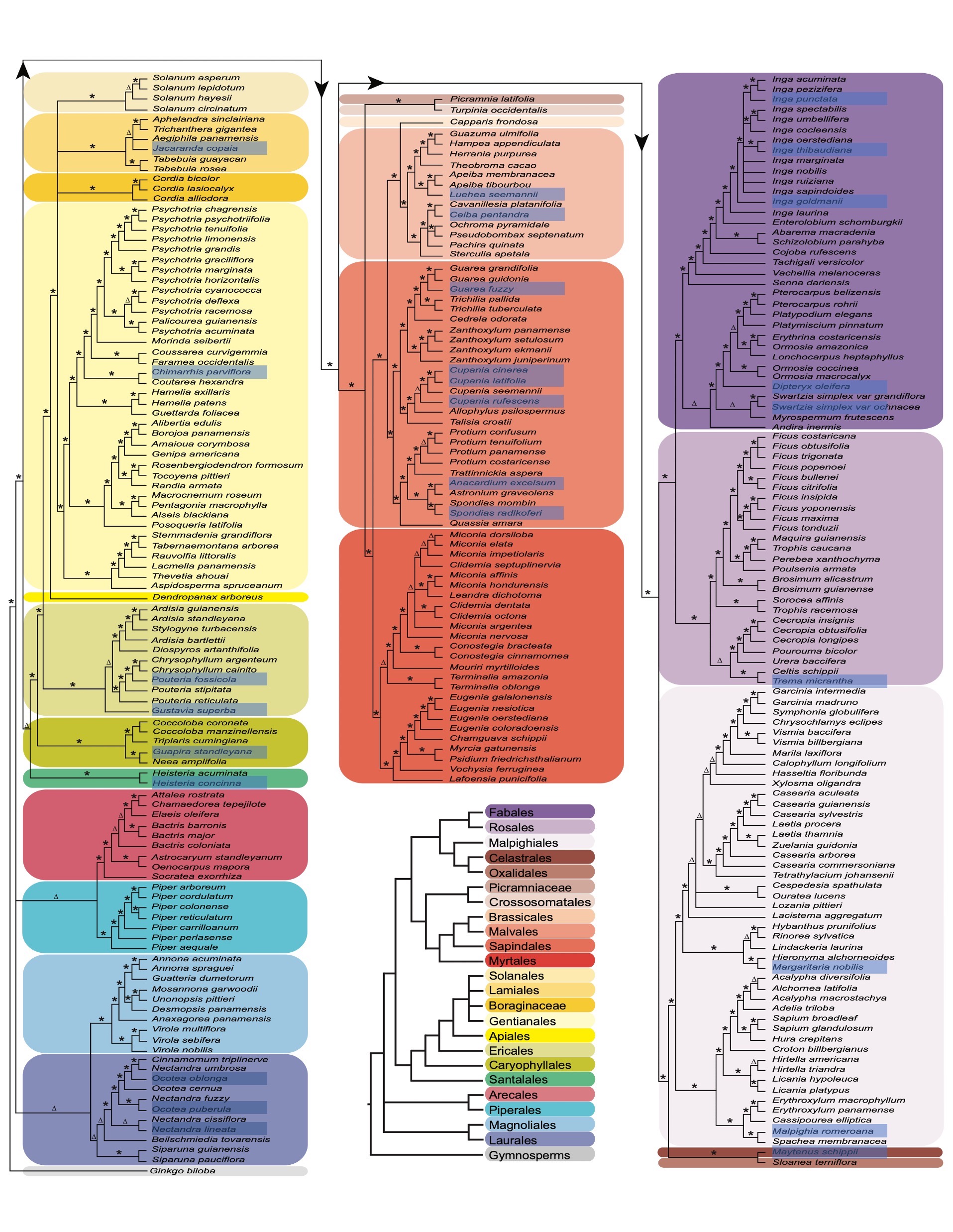

Supplement: Supplemental materials [file NIHMS1784086-supplement-Supplemental_materials.zip › FigureS1.jpg]
